# Medicine, Pathology and Therapeutics

**Published:** 1897-06

**Authors:** 


### Progress in Medical Science.

### MEDICINE, PATHOLOGY AND THERAPEUTICS.

Conducted by a member of the editorial staff.

### PROGNOSIS IN PNEUMONIA.

OSLER (*Amer. Jour. of the Med. Sciences*) shows that pneumonia is the most fatal of the acute infections of adults in temperate climates, the case mortality varying from 20 to 30 per cent. As to prognosis, age is very important ; in children, for instance, the mortality, as quoted in Wells's statistics, is 1.9 to 3.3 per cent., while over 60 years of age the mortality is 50 to 80 per cent. Negroes show a higher death-rate, as a rule, than whites. Kidney disease was found to be present in 25 out of 100 autopsies observed by the author. Healthy adults seldom succumb ; thus, in the German army the death-rate in 40,000 cases was only 3.6 per cent. Of the special points of prognostic importance, the degree of fever and the amount of lung-tissue involved are far less important than the toxemia. Toxemia has no constant relation to the extent of the lesion, and cases with profound toxemia show wide variations from the typical clinical picture, while the local features of the disease are often quite overshadowed. Such cases early show cerebral symptoms, and are met with especially in epidemics and among old patients. Sudden death may be due to the action of the specific toxins, which probably affect the heart-centers rather than the myocardium ; symptoms of collapse may set in within the first twenty-four hours and rapidly prove fatal. The amount of lung affected, even though extensive, is a much less potent cause of death than toxemia. The depressing action of the toxins on the cardio-respiratory centers and the weakening effect of pyrexia on the heart-muscle are important factors during the crisis, and help to explain the fact that venesection in pneumonia is not attended with such good results as in emphysema, morbis cordis and the like. Against toxemia, which is the important cause of the high mortality, there are at present no reliable measures at our disposal.

### PAROXYSMAL TACHYCARDIA.

A CHILD of eleven years was discussed by the London Clinical Society (*Med. Record*) on the 8th of January. The condition had existed for five years. The palpitation would come on suddenly and cease quite as suddenly after a period of from one to fourteen days, during which the pulse beat would be 240 to 260 per minute. At times there was slight cyanosis; urine scanty; no valvular disease; no lues. Changes in the myocardium were blamed for the affection, which seems to be rare in childhood. In the discussion which followed, the possibility of nutritive disturbance of the heart center in the medulla was suggested. It was also stated that in many instances, in which during life no heart affection was discoverable, an anatomical lesion of the myocardium would be made out at the autopsy. Disease of the nervous system does not explain paroxysmal tachycardia, since affections of the vagus itself do not give a pulse beat of over 150; the displacement of the abdominal organs likewise fails to explain the condition. As remedial measures, a vesicating plaster over the vagus, a combination of arsenic and bromide of ammonium, valerianate of zinc in beginning dose of two decigrams, and compression of the thorax and abdomen at the time of attack, were all recommended.

---

### UNPLEASANT EFFECTS OF TRIONAL.

Dr. F. Kaempffer (*Therapeutische Monatshefte*) relates the cases of four elderly persons suffering with cancer of the stomach or of the liver, with impaired nutrition, in whom fifteen grains of trional, instead of causing sleep, gave rise to a peculiar disturbance manifested by restlessness, anxiety, palpitation of the heart, and illusions, which lasted all night. In one of the cases a dose reduced to three-quarters of a grain produced a moderately sound sleep for six or seven hours; in the others, however, there was no change from lowering the dose, and an increase of it resulted in severer disturbance.—*N. Y. Med. Jour.*

---

### PERIODICAL PAROXYSMS OF HICCOUGH.

S. Feilberg (*Norsk Magazin for Lägeordenskab*) describes the case of a man, 42 years old, who suffered for several months from periodical paroxysms of hiccough, which entirely prevented his working and caused considerable suffering. After having searched in vain for the cause of the hiccough, the author finally found, at the base of the tongue, a tumor having three lobes, of which the central one, the largest, was about the size of a hazel-nut. These growths were due to a pronounced hypertrophy of the adenoid tissue at the base of the tongue, which caused, in addition to the hiccough, a continual sensation of fullness in the throat. There was also enlargement of the palatal tonsils, which were excised without producing any noticeable improvement. Ablation of the central lobe of the lingual tonsil caused considerable amelioration of the hiccough, which ceased entirely after the extirpation of the lateral lobes.

---

### ORDINANCE AGAINST COCAINE.

It is reported (*St. Louis Med. and Surg. Jour.*) that the victims of the cocaine habit have become so numerous in Chicago that an ordinance has been introduced prohibiting the sale of remedies for catarrh and other diseases, which contain cocaine. In the last two months over forty victims of the drug have appeared in the police courts and elsewhere. Several of them have been well-known men and women who say they were brought to their present condition by using catarrh cures.

---

### ATROPINE TO REDUCE THE TINNITUS AURIUM CAUSED BY THE USE OF QUININE.

Dr. Aubert has noted (*La Médecine Moderne*) that in three patients who had formerly suffered from tinnitus aurium due to quinine, the symptoms could at least be considerably diminished by adding atropine to the quinine. The amount of atropine prescribed by Dr. Aubert amounted to 0.0005 gramme ($\frac{1}{120}$ grain) daily.

---

### THE DIAGNOSIS OF TUBERCULOSIS.

A careful study of 100 tuberculous cases, (A. Mansfield Holmes, *Medical Record*,) including all stages of the disease, has shown conclusively that the law that brings about disintegration in a tuberculous patient brings about the same process at an earlier date in tuberculous leucocytes. In these cases I have found (*a*) marked disintegration going on in all varieties of leucocytes and (*b*) a great decrease in the number of young cells. These conditions are evidence that the tissue-forming power of these cells is imperfect or defective. Hence, when these conditions exist in cells, we may be absolutely certain that a similar condition already exists or soon will exist in the larger organism. Therefore, from the condition of the various cell tissues observed in these cases, I feel justified in making the following

*Deductions.*—First, it is possible to estimate the degree of the tuberculous condition. Second, it is possible to estimate the degree of the recuperative power. Furthermore, the phenomena upon which the foregoing deductions are based are as follows: (1) The degree of the tuberculous condition may be estimated by (*a*) the amount of deviation from the normal percentage of each variety of cells; and by (*b*) the amount of cell disintegration in each variety. (2) The degree of the recuperative power may be estimated by (*a*) the staining power of the nuclei; (*b*) the percentage of leucocytes with no evidence of disintegration; (*c*) a relatively high percentage of young cells; (*d*) the abundance of well-stained granules of the phagocytes; and (*e*) the abundance of eosinophile cells, rich in granules. I do not wish to give the impression that it is an easy task to interpret the phenomena presented in a specimen of tuberculous blood, and from them to reach a diagnosis. But it can be done. And when it is properly done, it furnishes a diagnosis based upon the fundamental principles of biology. Hence, from a study of the foregoing cases, I feel justified in claiming that the blood, aided by the microscope, together with a uniform and accurate technique, furnishes a means of making a positive diagnosis of the tuberculous condition early enough to allow of effective treatment.

---

### TUBERCULOSIS.

As MY view of the origin of tuberculosis (*Cincinnati Lancet-Clinic*) is at variance with established teaching, perhaps a simple statement of belief is necessary.

1. Tuberculosis is a constitutional disease, dependent largely on the evils of civilisation, and governed by the following law: The death-rate from tuberculosis is in direct ratio to suspension of atmospheric influence.

2. The suspension or abeyance of atmospheric influence may take place from within or from without. From without through impure or impoverished atmosphere; from within through defective lung tissue, original or acquired.

3. Abeyance of atmospheric influence, in whatever way induced, causes a depraved tissue or dyscrasia, through which the tubercle bacilli enter and grow.

4. The growth of tubercle bacilli, being dependent on the precedent state or condition of the individual, is never *per se* the primary cause of tuberculosis.

5. Tuberculosis may exist, though infrequent, in the absence of tubercle bacilli, but can never exist without the precedent state or condition.

6. The plant growth being a secondary condition or modifying influence in this most fatal disease, treatment directed against this growth must ever remain barren of permanent curative results and dangerous to the patient.

7. The precedent or primary condition being subject to law, and under control, tuberculosis is under control.

A question is anticipated. If tuberculosis be under control, why don't you control it? *Why don't you control it?*

The control of tuberculosis pertains to no one physician, only in pointing out the way. These seven propositions point the way, and on these the writer rests.

---

### FORMULÆ.

By H. E. SANDERSON, Ph. B., M. D.,
Assistant physician State Asylum for Insane, Stockton, Cal.

### LOCAL ANESTHETIC.

The following combination is said to be almost magical in its action:

| | |
|---|---|
| Chloroform | 12 parts. |
| Tinct. of aconite | 12 " |
| Tinct. of capsicum | 4 " |
| Tinct. of pyrethrum | 2 " |
| Oil of cloves | 2 " |
| Gum camphor | 2 " |

Dissolve the camphor in the chloroform and add the oil of cloves, and lastly the tinctures.—*Medical World.*—*Medical Age.*

---

### FRODIUM CICUTARIUM FOR UTERINE HEMORRHAGES.

An infusion of this plant, made from one part of the plant with twelve parts of water, is said to be more powerful than ergot. A dessertspoonful is given every two hours.—*Journal des Practiciens.* —*New York Medical Journal.*

---

### VULVAR VEGETATIONS.

| | |
|---|---|
| Pulv. savin | 1 gm. |
| Alum. dessicat | 25.0 āā |
| Hydrarg. chlorid. corros | 1.0 |

A more active application is the following:

| | |
|---|---|
| Pulv. savin | Equal parts. |
| Iodoform | |
| Acid salicylic | |

—*New York Medical Journal.*

### BITES OF FLEAS, MOSQUITOS AND BEDBUGS.

| | |
|---|---|
| Olei olivæ | 20 gm. |
| Ungt. styracis | 25 |
| Balsam. Peruvian | 5 |

—*Medical Record.*

### LUPUS ERYTHEMATOSUS.

| | |
|---|---|
| Liquor potassii arsenitis | 1 part. |
| Aquæ | 6 parts. |

Sig.—Apply twice daily.—*Gaz. Hebd.—Medical Record.*

### A CORRIGENT IN CHLOROFORM AND ETHER INHALATIONS.

Dr. E. Fraenkel (*Tag. des Operat. Gynäkol.*) recommends the following formula:

| | |
|---|---|
| Morphine hydrochloride | 2.0 gr. |
| Atropine sulphate | 0.2 " |
| Chloral hydrate | 3.5 " |
| Distilled water | 210.0 min. |

Sig.—Inject subcutaneously, from 1 to 1¼ grammes, shortly before anesthetisation.—*Centralblatt f. Chirurgie.*

### EUCALYPTUS IN STRYCHNINE POISONING.

A decoction of eucalyptus is of great value and may be used for lavage of the stomach in cases of strychnine poisoning.—*Exchange.*

### COLLODION FOR HEMORRHOIDS.

Dr. Samways recommends, in the *British Medical Journal*, the use of styptic collodion, applied on cotton-wool to the hemorrhoids each morning after defecation.

### THE VALUE OF CAMPHORIC ACID IN THE TREATMENT OF NIGHT SWEATS.

H. A. Hare (*Therapeutic Gazette*) says: In the January issue of the *Edinburgh Medical Journal* my favorite friend, Dr. Ralph Stockman, contributes a paper, in which he notes the value of camphoric acid in the treatment of night sweats, and quotes considerably from foreign literature on this subject in support of his own clinical experience.

The writer of this article has on several occasions, in papers which have been published, and every year to his class of medical students, emphasised the value of this remedy as an antisudorific.

His first experience was in the wards of St. Agnes' Hospital during 1890-91, where he found, as Dr. Stockman has, that it controlled the sweats of tuberculosis in the great majority of cases and did not produce any disagreeable symptoms whatever, such as are usually caused by atropine or other powerful antisudorifics. He also, in the first edition of his Text-book of practical therapeutics, published in 1891, spoke of the value of this drug for the purpose, and in *The Medical News* of April 4, 1891, he contributes a paper, in which he details two cases which have been markedly benefited by very moderate doses of the drug. He pointed out in this paper that twenty grains was usually quite sufficient to control the sweat, provided it was given early enough to be absorbed before the time of the sweat was reached; and he also pointed out, what other Continental observers had previously noted, that as much as sixty grains of this drug may be given without deleterious effect. A continued large employment of camphoric acid in the six years that have succeeded this report has still further confirmed his high opinion of this remedy; it will fail in some cases, but no remedy has been met with which in his hands so universally succeeds. It may be given in cachet, dissolved in whisky or brandy, or placed in dry powder upon the tongue and washed down with a little water or milk; but as it is slowly absorbed it should be given an hour or two before the time at which the sweat usually comes on. The writer has also found camphoric acid of great value in the treatment of idiopathic ptyalism as is sometimes met with in young children, as it controls the salivation without disordering the diges tion.

---

### TREATMENT OF TYPHOID FEVER.

So MANY inquiries have been received by us (*Memphis Medical Monthly*) and the Sisters of St. Joseph's Hospital, Memphis, relative to the treatment of typhoid fever, so successfully used by the former house-physician of that institution, Dr. Alfred Moore, that we reproduced the prescription from the March issue of the *Monthly*:

| | | |
|---|---|---|
| ℞ | Salol | ʒj. |
| | Thymol | xxxvj. gr. |
| | Bismuth subnitrat | ʒij. |
| | Muc. acaciæ | ℥ij. |
| | Syr. tolutan | ℥iv. |

M. S. Tablespoonful three times daily.

### CASES OF GALL-STONE.

RUTHERFORD MORISON, of Newcastle-on-Tyne, (*Universal Medical Journal*) emphasised (as Osler and he himself had previously done) the absence of pain as a prominent symptom in many of these cases. (1) A woman, aged 44, had suffered from jaundice for some months ; pain had been present only at its onset ; every time she rose she had a rigor and jaundice increased. There was a history of previous recurrent attacks of colic. On physical examination the liver was found enlarged, but no sign of distension of the gall-bladder could be made out ; at the operation the common duct was found blocked by a stone, the gall-bladder empty, contracted, and with dense, thickened walls. (2) A woman, aged 49, had suffered up to thirteen months previously from spasms of abdominal pain ; since then had lost flesh and noticed a tumor in the right side. On examination a movable tumor, the size of a cocoanut, was felt in the right hypochondrium, while at the operation a calculus was found blocking the cystic duct and enormously distending the gall-bladder. In this case there had been no jaundice. (3) A woman, aged 50, had suffered for two years from attacks of abdominal pain. Jaundice had supervened and was increasing rapidly, and a small tumor could be felt in the right hypochondrium. At the operation the gall-bladder was small and contracted, but there was a thin-walled diverticulum (the tumor felt), which contained a stone, as also did the common duct. An interesting comparison was drawn between the effects of complete and partial occlusion of the biliary passages on the gall-bladder, and those of analogous urethral obstruction on the urinary bladder. It was pointed out that in cases of obstruction of the common duct it was rare to meet with dilatation of the gall-bladder.

---

### A REPORT OF CASES OF SUN-STROKE DURING THE SUMMER OF 1896.

THE cases under consideration, reported by N. R. Norton, of New York, (*New York Med. Jour.*, March 6, 1897,) occurred in the wards of the Presbyterian Hospital, in the service of Dr. J. P. Thornley. During six days, from August 8th to 13th inclusive, about 110 persons were brought to the hospital suffering from the excessive heat. In the mild cases cold water was given, with which they sponged their heads and chest, and they were kept in a cool room and left the hospital in from fifteen minutes to twelve hours. The severer cases, with a temperature of 104° F. (40° C.) or over, numbered 61, with 9 deaths—a mortality of 14¾ per cent. In these hydrotherapy and skilled and careful nursing were the chief factors; the very frequent recording of the temperature, enabling the baths to be given at the earliest and, therefore, most valuable time; the use of the ice tub-bath, with constant and general friction of the entire surface, thus reducing the temperature in the shortest possible time, and being stimulating rather than depressing; the use of the same bath for all severe secondary elevations of temperature, and for the minor elevations sponge-baths of ice-water or of water at from 70° to 80° F. (21.1° to 26.7° C.), depending upon the individual case; and the repetition of these baths whenever the temperature is high enough to make them seem advisable.

All other means seemed entirely inadequate.

The severity of the epidemic, the large number of cases occurring in so short a time, and the crowding consequent to such an emergency make the low death-rate of 9 in 61 cases (11 not tabulated), or 14$\frac{3}{4}$ per cent., extremely gratifying.

In this hospital, from August, 1888, to April, 1896, there were 22 cases of insolation with temperatures corresponding to the arbitrary standards adopted above, with 9 deaths—a mortality of 41 per cent. At the Pennsylvania Hospital, in the summer of 1887, there were 31 cases, with 12 deaths—a mortality of 35 per cent. A report by Swift, of 150 cases, records 78 deaths—a mortality of 52 per cent.

A comparison of these records shows the importance of noting, if possible, any points in the treatment of cases in the epidemic of the past summer which could, in any way, account for the marked difference in mortality.

---

### SOME CASES OF ASTASIA-ABASIA.

Charles Petren reports (*Hygeia*, Vol. LVIII., No. 11, pp. 515 to 547, 1896,) several cases, of which the first was an unmarried woman, 28 years of age. This he considers as one belonging to the chorea-like variety.

*Case 2.*—A single woman, 22 years old; also a case of chorea-like abasia.

*Case 3.*—Unmarried woman, 47 years old—a case which showed unmistakable partial abasia, complicated with hysterical stigma.

*Case 4.*—Man, 69 years of age, which the author considers as one of trepidating abasia.

With regard to the nature of the disease, it seems to the author that any anatomical alteration in the spinal cord as cause of the disease must be definitely excluded, and that a similar alteration in the brain may be regarded as improbable. Abasia must, therefore, be considered as a dynamic disease. Walking being, in all probability, a function of the brain, the author furthermore believes that abasia is due to a disorder of that organ, and that even that disorder is of a psychical nature, the more so since the motor derangement is manifestly systemic and limited to a certain system of movements. He cites a case as an example of a systemic paralysis, which is quite the contrary of abasia. For the time being no other conception seems to the author to be legitimate than to consider the abasia as a manifestation of hysteria—that is to say, the cause of the abasia must be looked for in that portion of the mental life which is distinct from consciousness, or, in other words, in the non-dominion of the conscious part of the life of the soul over the psychical processes.

